# An evolutionary machine learning algorithm for cardiovascular disease risk prediction

**DOI:** 10.1371/journal.pone.0271723

**Published:** 2022-07-28

**Authors:** Mohammad Ordikhani, Mohammad Saniee Abadeh, Christof Prugger, Razieh Hassannejad, Noushin Mohammadifard, Nizal Sarrafzadegan

**Affiliations:** 1 Faculty of Electrical and Computer Engineering, Tarbiat Modares University, Tehran, Iran; 2 Institute of Public Health, Charité—Universitätsmedizin Berlin, Cooperate Member of Freie Universität Berlin and Humboldt-Universität zu Berlin, Berlin, Germany; 3 Interventional Cardiology Research Center, Cardiovascular Research Institute, Isfahan University of Medical Sciences, Isfahan, Iran; 4 Hypertension Research Center, Cardiovascular Research Institute, Isfahan University of Medical Sciences, Isfahan, Iran; 5 Isfahan Cardiovascular Research Center, Cardiovascular Research Institute, Isfahan University of Medical Sciences, Isfahan, Iran; 6 School of Population and Public Health, Faculty of Medicine, University of British Columbia, Vancouver, British Columbia, Canada; Menoufia University, EGYPT

## Abstract

**Introduction:**

This study developed a novel risk assessment model to predict the occurrence of cardiovascular disease (CVD) events. It uses a Genetic Algorithm (GA) to develop an easy-to-use model with high accuracy, calibrated based on the Isfahan Cohort Study (ICS) database.

**Methods:**

The ICS was a population-based prospective cohort study of 6,504 healthy Iranian adults aged ≥ 35 years followed for incident CVD over ten years, from 2001 to 2010. To develop a risk score, the problem of predicting CVD was solved using a well-designed GA, and finally, the results were compared with classic machine learning (ML) and statistical methods.

**Results:**

A number of risk scores such as the WHO, and PARS models were utilized as the baseline for comparison due to their similar chart-based models. The Framingham and PROCAM models were also applied to the dataset, with the area under a Receiver Operating Characteristic curve (AUROC) equal to 0.633 and 0.683, respectively. However, the more complex Deep Learning model using a three-layered Convolutional Neural Network (CNN) performed best among the ML models, with an AUROC of 0.74, and the GA-based eXplanaible Persian Atherosclerotic CVD Risk Stratification (XPARS) showed higher performance compared to the statistical methods. XPARS with eight features showed an AUROC of 0.76, and the XPARS with four features, showed an AUROC of 0.72.

**Conclusion:**

A risk model that is extracted using GA substantially improves the prediction of CVD compared to conventional methods. It is clear, interpretable and can be a suitable replacement for conventional statistical methods.

## Introduction

Cardiovascular diseases (CVDs) are the leading cause of morbidity and mortality worldwide [1, 2]. CVDs impose heavy social and financial burdens, including direct costs of diagnostic equipment and specialists as well as other indirect costs resulting from reduced quality of life, loss of productivity, and morbidity [3]. Moreover, the diagnostic equipment is primarily available in specialized hospitals within large cities; thus, small towns and suburban areas suffer from the shortage or lack of such services [4]. Therefore, there is a great necessity to develop computational methods for estimating the occurrence of CVD events in clinical practice [5]. The computational methods can help identify high-risk individuals and motivate them to change their behaviors for preventive medicine purposes [6]. These CVD models are categorized into four groups based on their risk scores outputs: 1. If-Then models (e.g. Framingham [7] and PROCAM [8]) 2. Formula-based models (e.g. Reynolds [9]), 3. ML models (e.g. Random Forest and Deep Learning), and 4. chart-based model (e.g. PARS [10], SCORE [11] and WHO [6]). These models are either limited in terms of accuracy or interoperability.

In recent years, a broader concept known as explainability has emerged, which in some contexts has been denoted as eXplanaible Artificial Intelligence (XAI) [12]. XAI is artificial intelligence (AI) in which the results of the solution can be understood by humans [13].

In this study, CVD risk was predicted based on a novel method using a transparent and interpretable ML model, which could be understood and accepted by the medical community. To strike the right balance between interpretability and prediction accuracy, an evolutionary model is utilized, which benefits from the advantages of both ML and chart-based models. The resulting risk assessment model for estimating CVD occurrence in the Iranian population is vital to developing national prevention programs. This effort is the first explainable CVD model, the eXplanaible Persian Atherosclerotic CVD Risk Stratification (XPARS).

The results of XPARS improve upon ML and, statistical methods with regard to interpretability and accuracy. The WHO, SCORE, and PARS models were used as the baseline for comparison due to their chart-based systems. The proposed method significantly improves the accuracy of estimating the risk of CVD compared to the previous chart-based models, and besides, it is quite clear and interpretable.

## Methods

### Study population

The database of the Isfahan Cohort Study (ICS) in Iran was used for this project. The baseline survey was conducted in a representative sample of the Iranian adult population (n = 6,504) aged 35 to 84 years. Study participants were followed-up over 10 years from 2001 to 2011 or until they experienced a CVD event. According to a report presented by trained staff (e.g. registered nurses, specialists and general practitioners), and relying upon a standardized questionnaire, none of the participants had a history of chronic diseases. However, 181 participants with a history of myocardial infarction, stroke, or heart failure at baseline were excluded. The study was conducted after obtaining written informed consent, as described previously [10].

### Outcomes

The primary outcomes were fatal and non-fatal CVD events, including sudden cardiac death, unstable angina, myocardial infarction, and stroke. A complete structured interview and basic examination, including blood sampling, were performed at the beginning of the study in 2001 and then repeated in 2007 and 2011 using the same methods. Every two years, a phone interview was conducted, and the staff were sent to their residences in case of unreachability [10, 14].

### Proposed method

Information-Gain and Gain scales were applied to select and rank the most significant features. In addition, the forwarding method was used to select a subset of compatible features. There are two types of features: continuous (age, cholesterol, blood pressure (BP)) and dichotomous (sex, waist to hip ratio (WHR), family history (FH) of CVD, diabetic, and smoker). The chart-based models were divided into two categories based on the inclusion of cholesterol among the modelled features.

**Fig 1:**
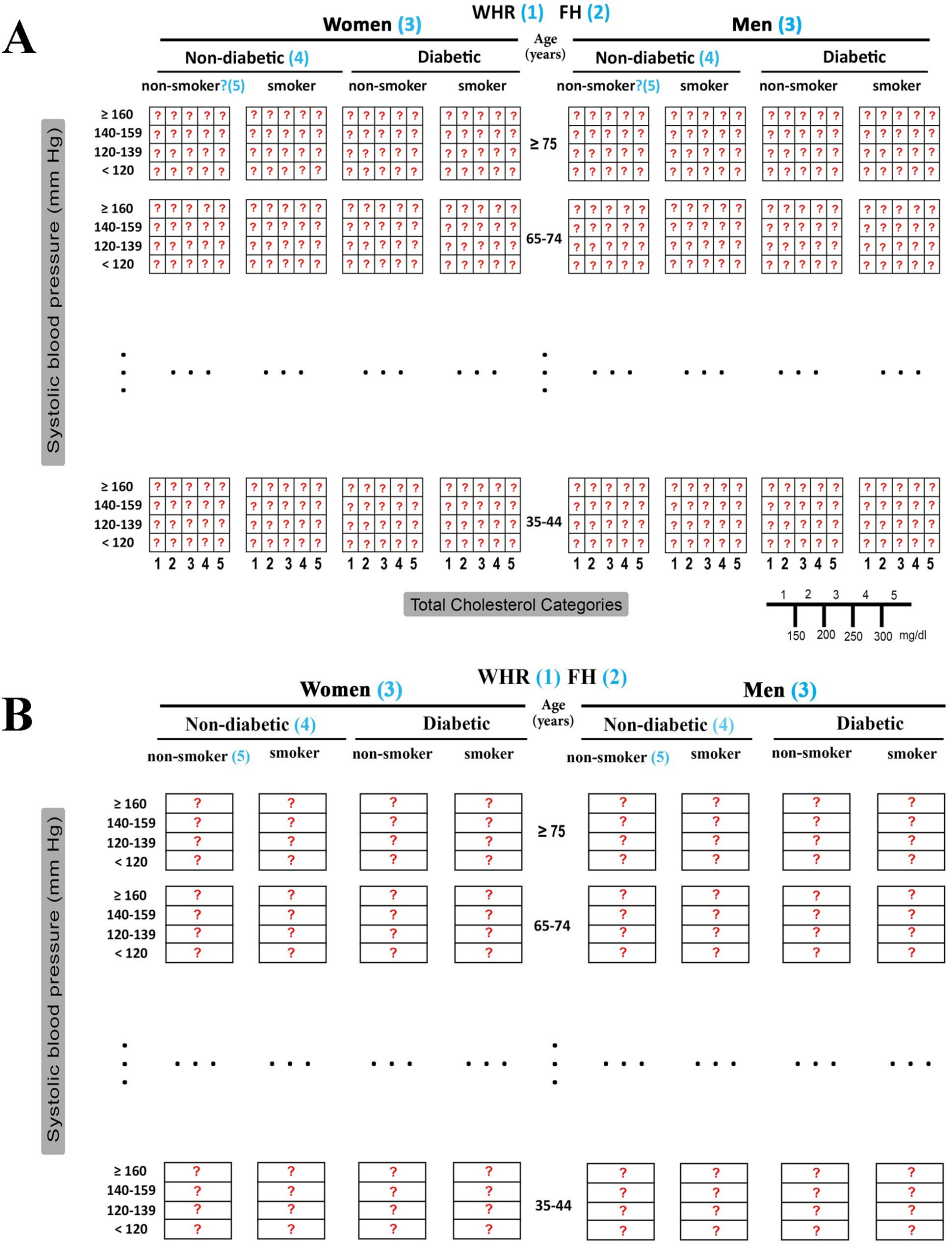
Chart-base representation of CVD risk score. (A) Two-dimensional (2D) representation, and (B) One-dimensional (1D) representation. Red question marks present CVD risk scores. In this example, the features were WHR (1), FH of CVD (2), sex (3), diabetic (4), and smoker (5). The row in each block shows the BP, which was grouped into four classes. (A) It is composed of 2D blocks, where each column represents cholesterol categories. Age was categorized into five groups.

The GA requires a representation that describes the problem states. Two types of CVD chart-based models were proposed based on the use of cholesterol as a feature, leading to one- and two-dimensional representations **(Fig 1).** The two-dimensional (2D) representation, which combines BP and cholesterol levels, is more widely used and depicted in the figure (**Fig 1A**). The chart is composed of same-sized blocks, each representing four categories for BP and five for cholesterol, making each block a 4×5 matrix.

The one-dimensional (1D) representation is the approach without cholesterol, in which each block is a 4×1 matrix representing the assessed risk at different BP intervals. The second representation is less common; hence, this study focuses on the first representation unless mentioned otherwise.

GA is an optimizer inspired by natural evolution, where proposed solutions evolve to get closer to the optimal. Each step in the evolution of the solution is inspired by an equivalent step in natural selection. For example, a pair of solutions are matched to produce a combined solution; the matched pair are called parents, and the solution is called a child.

In line with the evolutionary language, each proposed solution in GA is called a chromosome. Each chromosome represents a point in the search area consisting of a fixed number of genes (scores) [15]. Chromosomes are improved in each generation by matching and combining existing chromosomes in a process called reproduction.

**Fig 2:**
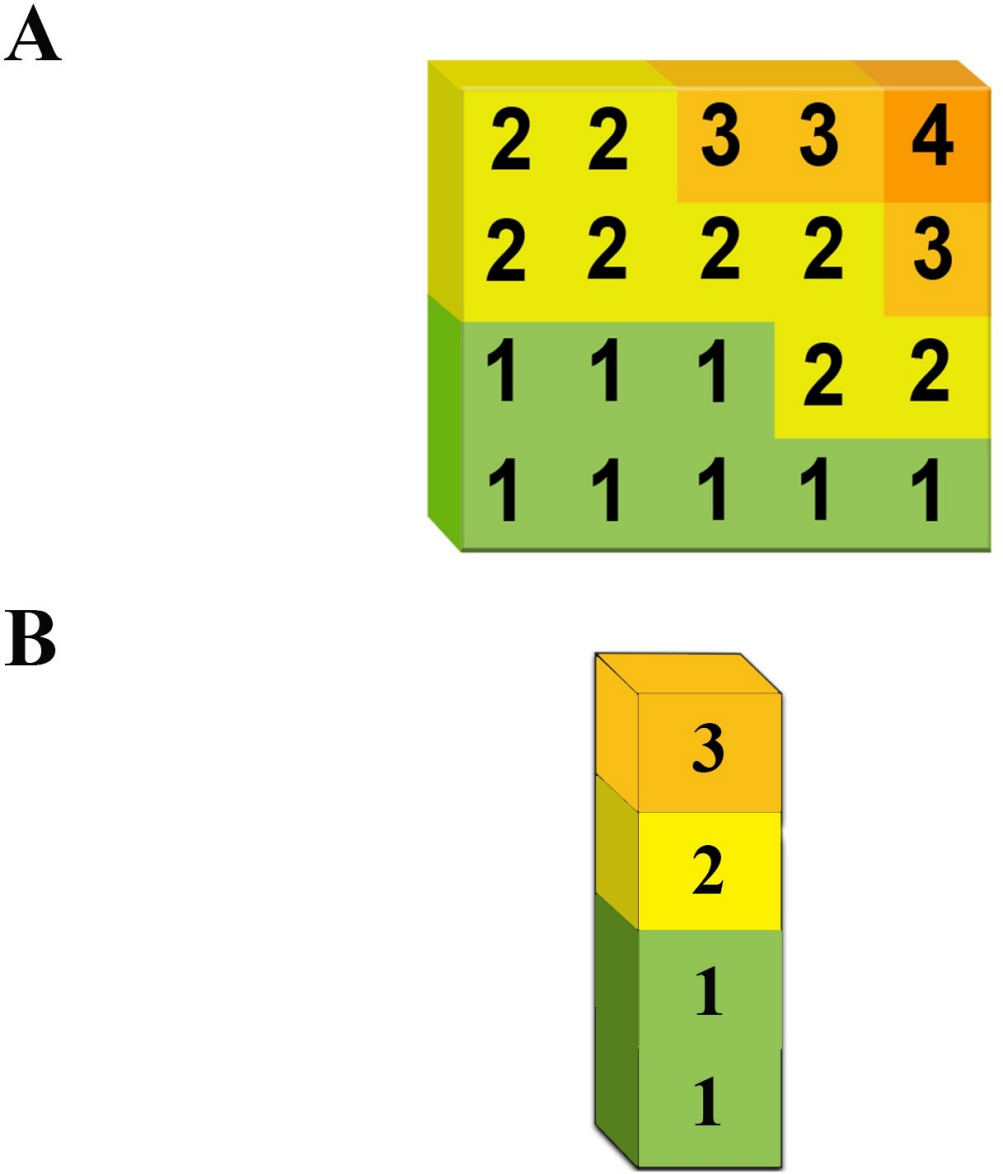
Chromosome representation in (A) two-dimensional (2D) representation, and (B) one-dimensional (1D) representation. A chromosome is a 4×5 matrix in 2D representation. Each value on the matrix is called a gene; genes increase from left to right and decrease from top to bottom. (B) A chromosome is a 4×1 matrix in 1D representation. The genes decrease from top to bottom.

For modelling the risk score, the chromosome was defined to be a heart disease matrix (a block in **Fig 1**). A chromosome is considered valid if genes increase from left to right in the rows decrease from top to bottom in the columns **(Fig 2A)**. In the 1D representation, cholesterol was dropped from the blocks, making each block a column of different BP categories. **(Fig 2B)**.

Population initialization is a crucial task in evolutionary algorithms because it can affect the convergence speed and accuracy of the final solution. Therefore, survival analysis was used to generate the initial population of chromosomes. The blank chart was filled using Cox regression, similar to Sarrafzadegan et al. analysis for the PARS model [10].

A fitness function is an objective function which evaluates how close a solution is to the desired solution. AUROC was selected as the fitness function, which is used to determine the overall performance of a solution.

The next step in GA is selection, which involves choosing chromosomes that are to be combined to produce the next generation of chromosomes in a process called crossover. The roulette wheel selection function was used to pick the appropriate chromosomes for the crossover operations. The probability of choosing an individual for breeding the next generation is proportional to its fitness; the better the fitness is, the higher the chance of being chosen.

The population of chromosomes in each generation are bred in a process called reproduction. Reproduction is comprised of two main steps: crossover and mutation. Crossover is a genetic operator used to combine the genetic information of two chromosomes (parents) to generate new offspring. A crossover operator exchanges gene sequences between two chromosomes with a probability of producing all combinations, a set of children. The mutation step introduces random changes to the genes.

To perform the crossover, gene subsets of different lengths were selected from the first parent. This starts by selecting the first gene from the first parent and the rest of the gene sequence from the other one and combining them, then the first two genes from the first parent and the rest from the other parent and combining, etc. Doing this, all the children from combining an initial part of the first parent’s gene sequence with the last part of the other parents’ sequence were found. For every combination, the validity condition was examined before considering it a child. The same procedure was performed by replacing the parents, combining the initial part of the second parent’s sequence with the latter part of the first parent genes.

**Fig 3:**
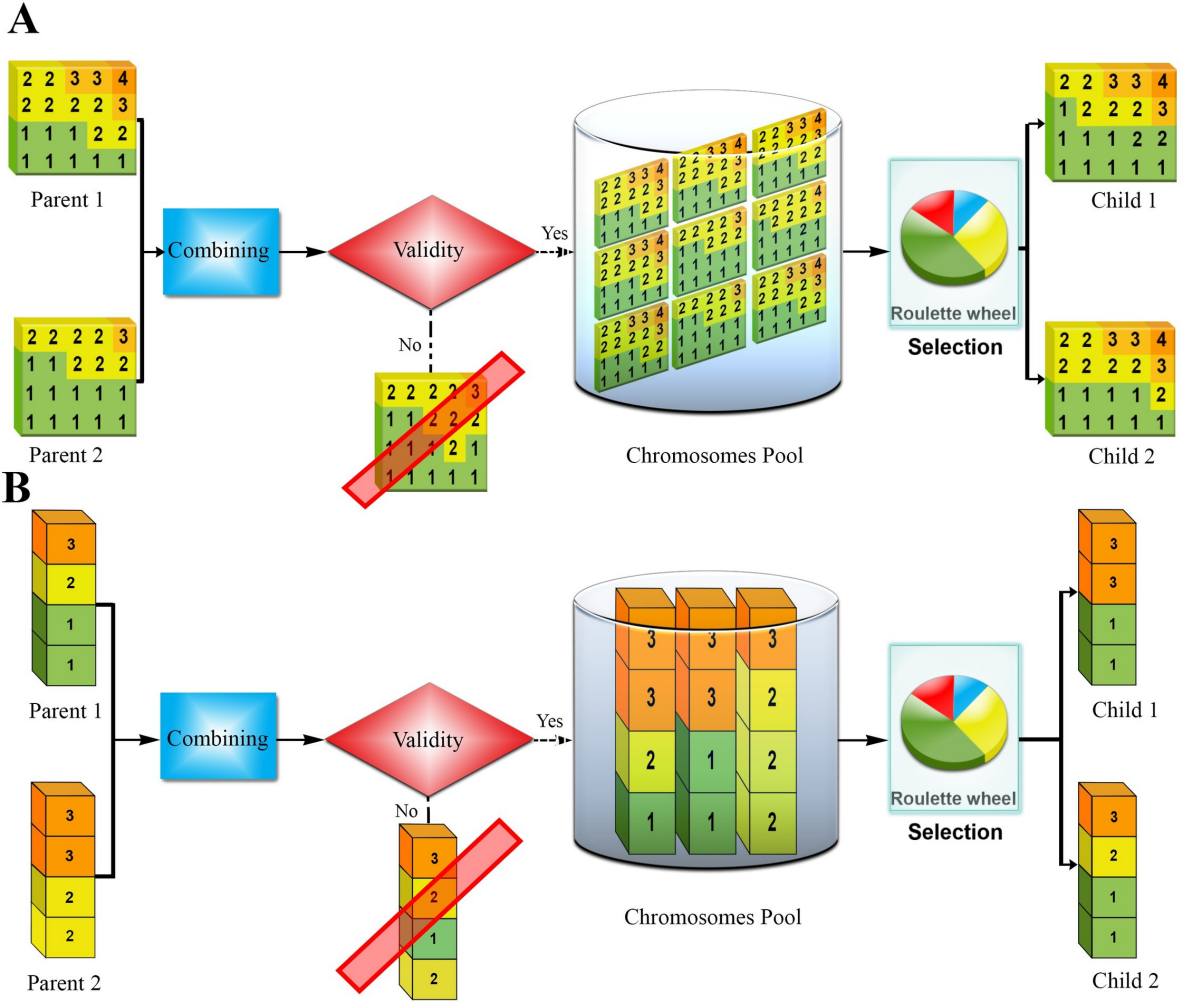
Crossover operations (A) 2D crossover, and (B) 1D crossover. **Step 1:** Combine parents’ chromosomes. **Step 2:** Check the validity of the resulting combination. **Step 3:** Obtain the pool of unique offspring. **Step 4:** Select two children by the Roulette Wheel selection.

After removing the duplicates, the resulting offsprings were placed in a pool of chromosomes. Among the children, two were extracted as final surviving chromosomes based on roulette wheel selection, the same function used for crossover. As a result, from every two-parent, two children were extracted for the next generation **(Fig 3A)**. The children’s selection step not only helped improve children’s fitness but also kept the population of chromosomes steady. The same method was used for 1D representation **(Fig 3B)**.

**Fig 4:**
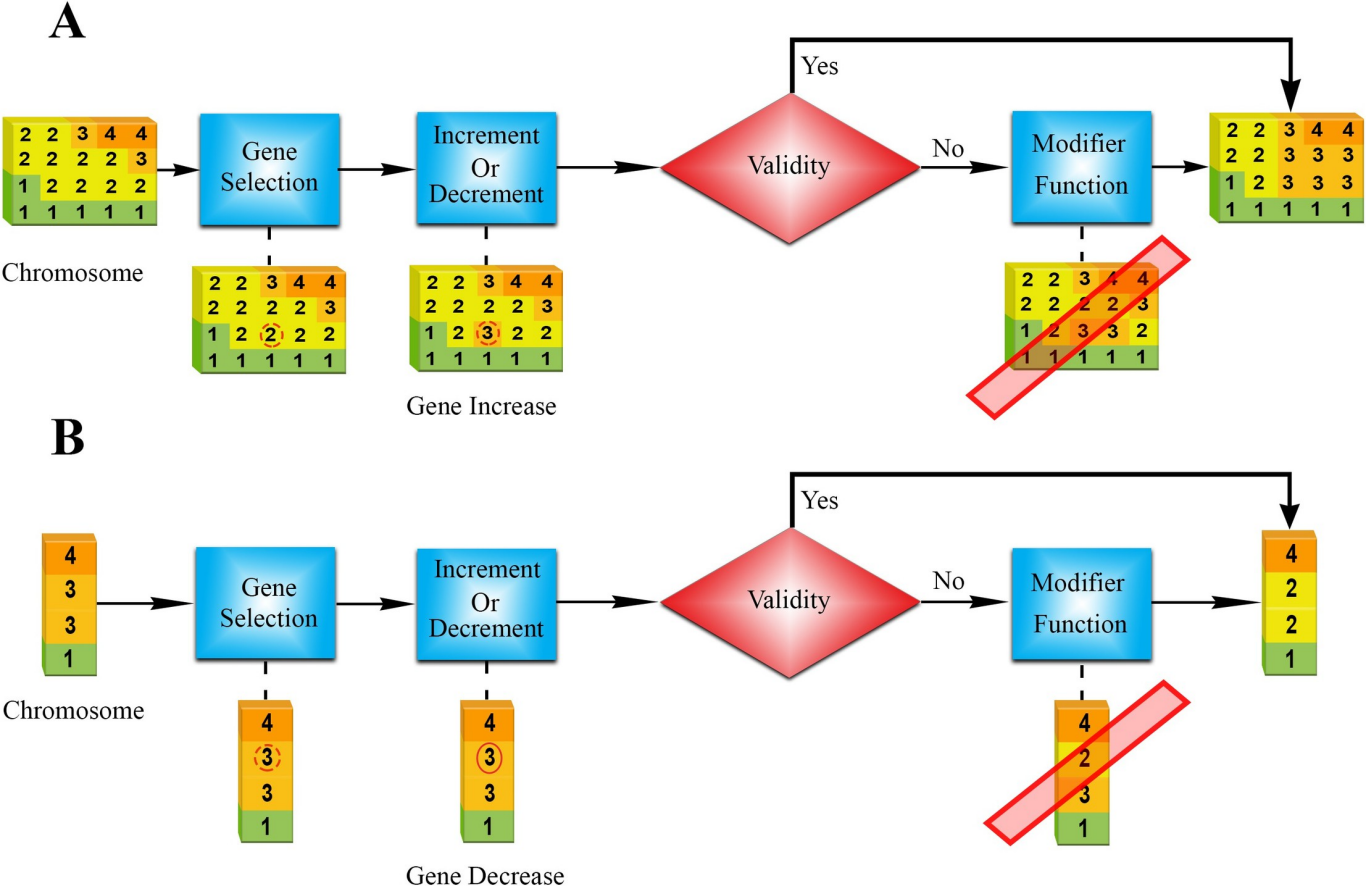
Mutation operation (A) 2D mutation, (B) 1D mutation. **Step 1**: Randomly select a gene. **Step 2**: Either increase or decrease the selected gene with the same probability. **Step 3:** Check the validity of the chromosome, and if invalid, send it to the modifier function. **Step 4**: The modifier function fixes the chromosome.

After the crossover operation, chromosomes are mutated before reaching the next generation. The mutation operator was applied to the children resulting in random changes in one or more genes. The operator randomly selects a gene from the chromosome and either increase or decreases it by one unit, with the same probability. The resulting mutation was checked for validity, and if found invalid, it was fixed by a modifier function (by recursively changing the chromosome until it became valid). This representative process is shown in (**Fig 4).**

The next generation of GA would be chosen from parents and their offspring, determined by a process called replacement. The replacement function for the risk score model was based on keeping the best chromosome (elitism) as well as a random selection of parents and children.

GA was applied to each chromosome (block) in the CVD chart, so the final solution for the whole chart involved iteratively applying GA to each chromosome, one by one. At each step, a chart’s chromosome was inputted to the algorithm and was replaced by the resulting solution, the outputted chromosome. Accordingly, a round of updates involved replacing every chromosome in the chart with corresponding solutions. The same process was repeated several rounds until we reached convergence throughout the chart. The ordering of updates was determined by the size of data in the block corresponding to the chromosome, starting from the chromosome with the largest number of observations. As a result, a chromosome that contained more data had a larger impact on AUROC.

The output of previous steps was introduced to a modifier function. The modifier function calculated the ratio of people who had a positive class label to the total number of people covered in each cell of the chromosome matrix. To maintain the chart order and chromosome validity, the gene was increased if this ratio was higher than 50% and was decreased otherwise.

All models were validated using 10-fold cross-validation. The data was divided into 10 segments in a way that the distribution of the class label is similar across segments. Of these divisions, a single one was retained as the validation data used for model testing, and the model was fit using the remaining 9 sections (the training data). This process was repeated 10 times, each sample being used exactly once as the validation data. In the end, the average over these ten models was reported. Using all observations for both training and validation, cross-validation leads to a more accurate evaluation of model performance compared to simply dividing the data into training and validation sets.

### Risk chart

As discussed, the calculated CVD risk scores are called XPARS. They are presented in an easy-to-read chart in a similar format as the PARS model. The CVD probabilities are color-coded in the risk chart, using the ranges “≤ 1%, 2%, 3%–4%, 5%–9%, 10%–14% and ≥15%”. The Python programming language and its libraries were utilized for modelling and statistical analysis.

### Risk factor variables

The chart predicts the 10-year CVD incidence based on the variables age, sex, BP, WHR, FH of CVD, diabetes, smoker, and cholesterol. BP was categorized into four groups: (1) <120, (2) 120–139, (3) 140–159, and (4) ≥160 mm Hg. The high waist to hip ratio (WHR) was ≥ 0.80 in women and 0.95 in men. The subject was identified as a person with diabetes if their Fasting Blood Sugar (FBS) was ≥ 126 mg/dL, or 2-hour plasma glucose was ≥200 mg/dL, or the patient was receiving anti-diabetic treatment. The “smoker” variable includes current smokers. Cholesterol was also classified into five groups based on the National Adult Cholesterol Education Program: (1) <150, (2) 150–200, (3) 200–250, (4) 250–300, and (5) ≥ 300 mg/dl.

## Results

From the 5432 survey participants who were non-CVD at the baseline, there were 705 cases of CVD during the 10-year follow-up period. There were fewer women than men (51.3% F, 48.7% M) in CVD cases, while there were only slightly more women than men in non-CVD cases. Considering the various predictors and their interactions in a multivariate Cox regression, significant predictors of CVD events were age, sex, WHR, BP, cholesterol, diabetic, smoker, and FH of CVD [10] (**S1 Table in S1 File**).

Two representative 1D and 2D models were selected for exposition, the 2D model with eight features and a 1D model with four features. These two were selected from a larger pool of model variations based on different subsets of the features. The variations were selected using the forward feature selection method. Age, BP, and cholesterol were the common features across all 2D representations. On the other hand, age and BP only were the common features across 1D representations.

In all variations of 1D and 2D models, AUROC was higher than 0.70.

In addition to AUROC, which measures prediction accuracy, interpretability of a model is also quantified so that models can be compared considering both criteria. The explainability measure we report for representation is the total number of cells in the chart. The fewer the total number of cells in a chart is, the easier it is to read the chart by the human user.

**Fig 5:**
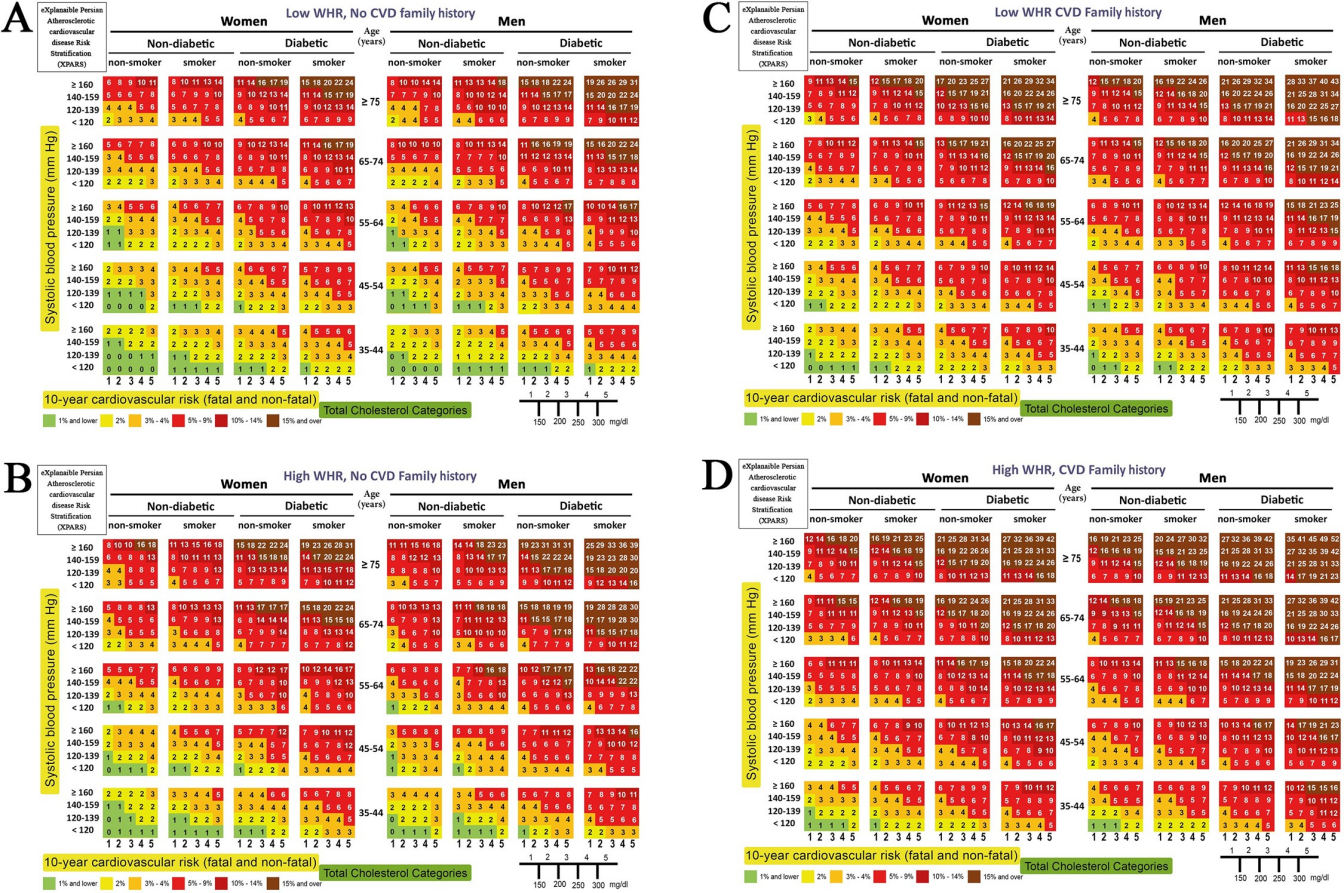
XPARS with eight features: Charts for prediction of 10-year risk of fatal and non-fatal CVD in the ICS population by sex, age, BP, smoker, diabetic, and cholesterol. (A) Low WHR and no FH of CVD, (B) High WHR and no FH of CVD, (C) Low WHR and FH of CVD, (D) High WHR and no FH of CVD.

The XPARS with eight features chart predicts the 10-year risk of fatal and non-fatal CVD by sex, age, BP, smoker, diabetic, cholesterol, WHR, and FH of CVD (**Fig 5**). The resulting chart has 3200 cells and leads to an AUROC of 0.76.

In XPARS with eight features, AUROC for the training data (training AUROC) is 0.80 However, the cross-validated AUROC is 0.76.

The 2D representation for XPARS involves 160 chromosomes, each corresponding to a partition of the data and represented by a block in the chart. Out of 160 partitions, only 107 of them have available data. The method starts from the chromosome with the largest corresponding data size and applies GA on all 107 blocks in each round, in the process described in the methodology section. By construction, at the beginning of the process, AUROC for XPARS is the same as the one for the PARS model (AUROC = 0.74 [10]). At the end of the first round of GA application, this value was increased to 0.80, and stayed at this level in the second round.

**Fig 6:**
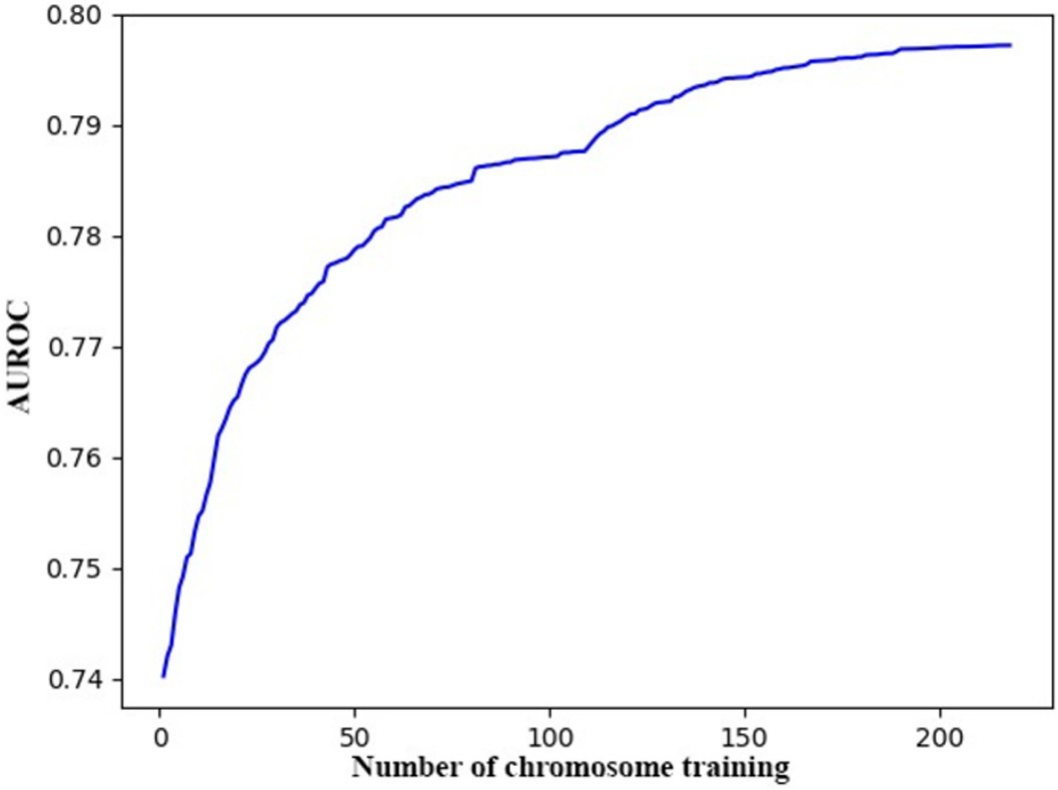
AUROC improvement with training (XPARS with eight features). The figure shows the improvement in training AUROC as it is trained with two complete rounds of non-empty chromosomes. The model used is XPARS with eight features, and the number of chromosomes with available data is 107 out of a total of 160 chromosomes. The process starts with an AUROC of about 0.74, while at the end of the first round of GA application, it raised to 0.80. After the second round, of training each of 107 chromosomes AUROC converged to 0.80.

The AUROC increased most in response to the initial chromosomes with the largest number of observations. But as more chromosomes were trained, AUROC responded less to training, especially as the later chromosomes with fewer data points came. As shown in Fig 6, the AUROC almost flattens at the end of the first round of training (at the 107^th^ chromosome) but picks up slightly in the second round as it is retrained with the chromosomes with the largest data points. The gain in the second round is only under about 0.01, and it plateaus fast. Hence, the process was stopped at two rounds, as there was no further gain from additional rounds **(Fig 6)**.

**Fig 7:**
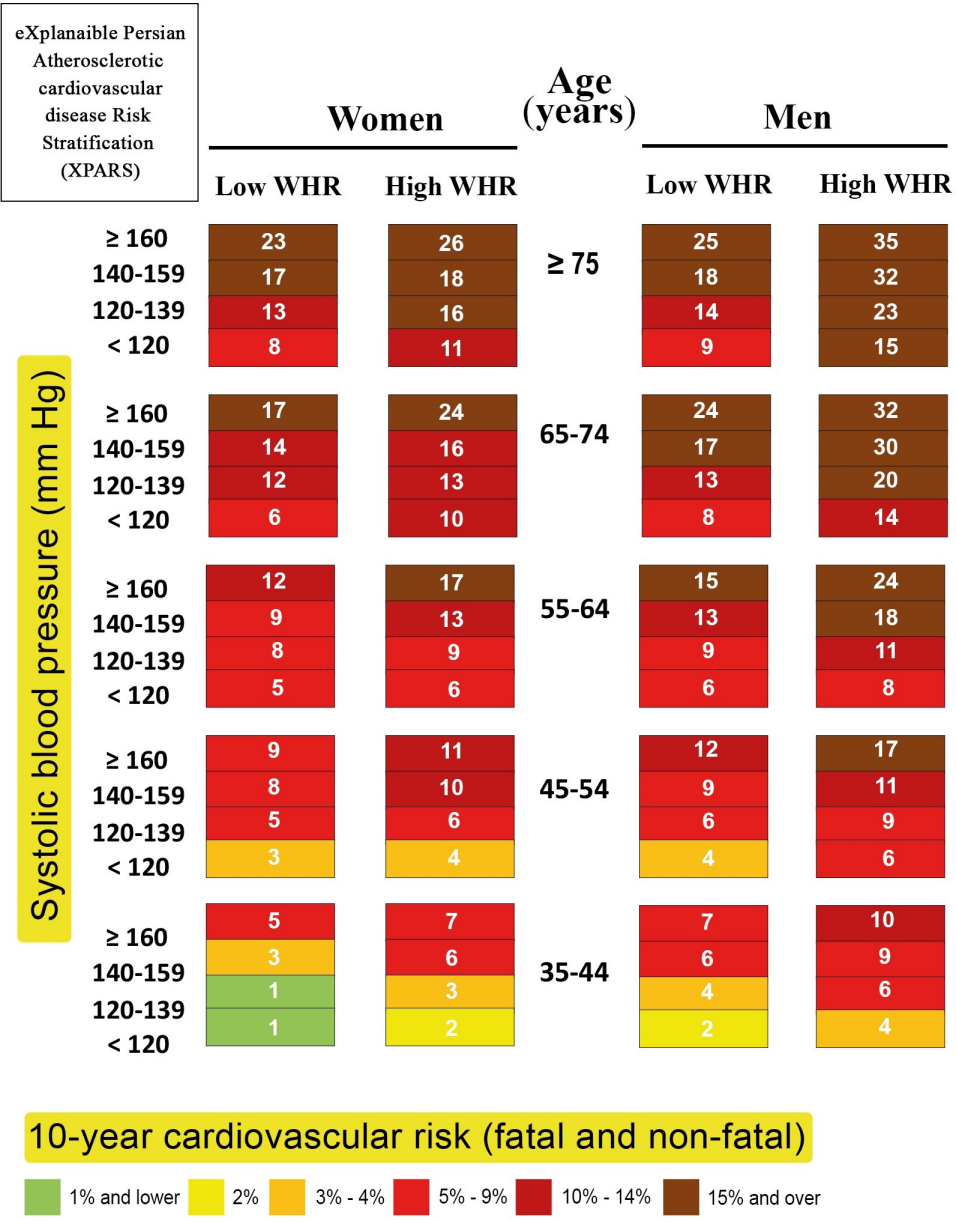
XPARS with four features: Charts for prediction of 10-year risk of fatal and non-fatal CVD in the ICS population by sex, age, BP, and WHR.

Moreover, XPARS with only four features were considered, which is much easier to use given the simpler chart and no need for lab-based cholesterol measurement. CVD risk score was estimated based on age, sex, BP, and WHR. The resulting chart has only 80 cells, leading to an AUROC of 0.72 **(Fig 7)**.

**Fig 8:**
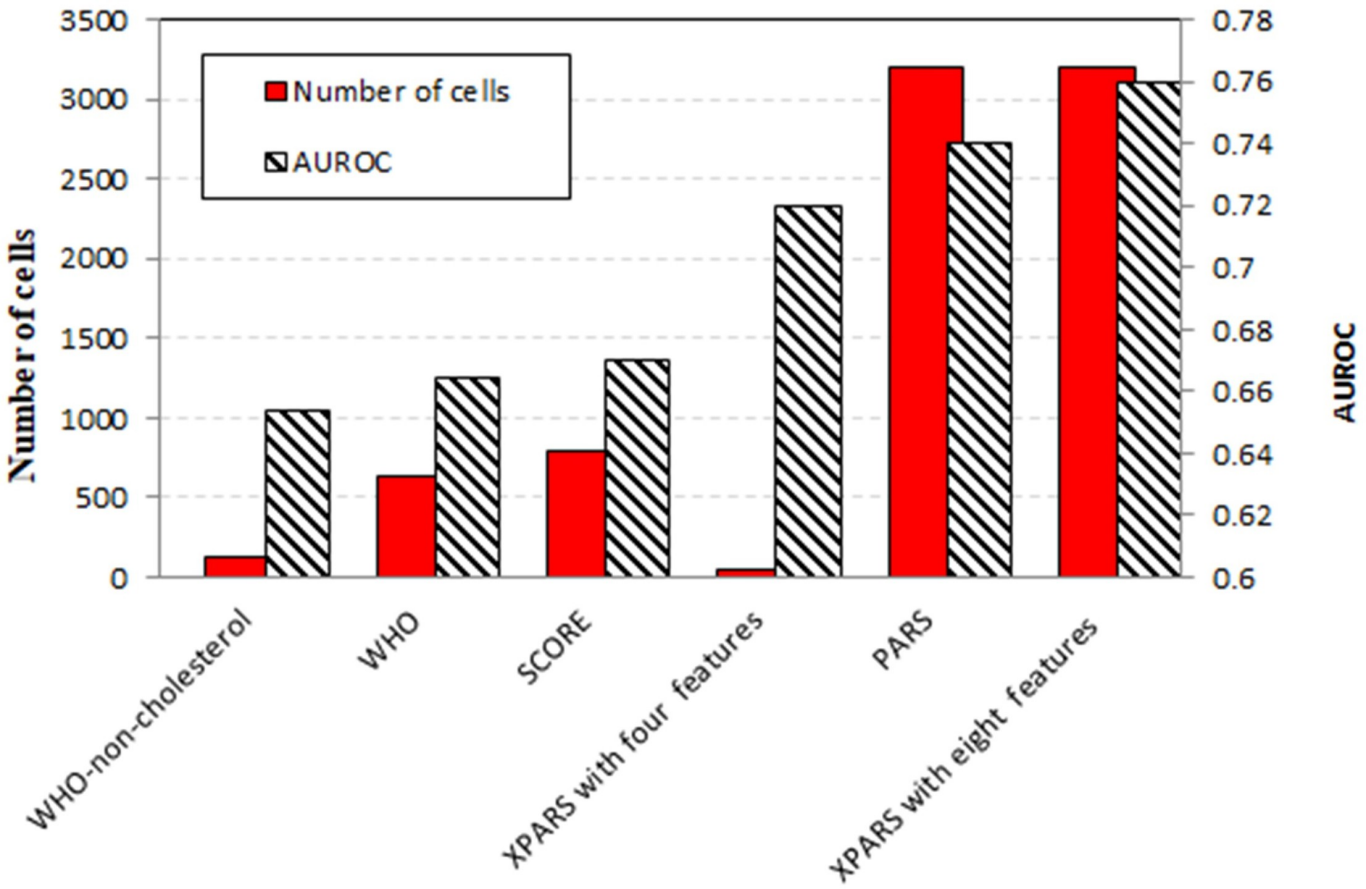
Comparison of interpretability and predictive accuracy of chart-based models. XPARS provides improvement to chart-based models in terms of both interpretability and prediction accuracy. The interpretability of models is measured based on the number of cells in the chart, while predictive accuracy is based on AUROC. XPARS, with four features, is the most interpretable without sacrificing accuracy by much. It improves the most interpretable previous model, non-cholesterol WHO, in terms of both interpretability (80 vs. 128) and AUROC (0.72 vs. 0.65). XPARS with eight features has a 2% higher AUROC compared to the PARS model, the most accurate previous model, given the same chart size.

The proposed method can create more accurate and interpretable models compared to other methods. The XPARS models were compared with other models based on the ICS dataset. In terms of interpretability, the PARS model is the most complex, with 3200 cells and AUROC of 0.74, while the 1D representation of XPARS is the most explainable. XPARS, with only four features and 80 cells, reaches an AUROC of 0.72 while being even less complex than the WHO non-cholesterol model with 128 cells, the simplest similar model **(Fig 8).**

In terms of prediction accuracy, XPARS with eight features outperformed previous chart-based models. The PARS model had the largest accuracy among those, where it attains an AUROC of 0.74 with eight features. Using the same features and, as a result, the same number of cells, XPARS could improve the AUROC of PARS to 0.76.

XPARS models were also compared to non-chart models, all fit based on the ICS data. **Table 1** provides an overview of comparing AUROC. XPARS could achieve larger AUROC compared to If-else and ML models. Among If-else models, the better-known FRAMINGHAM and PROCAM were used models for comparison. These models reached AUROC of more than 0.63 and 0.68, respectively.

**Table 1:** Results of the implement of popular models on ICS datasets.

Model type	MODEL	AUROC
If-else	PROCAM	0.683
	FRAMINGHAM	0.633
Machine learning	Classification via Regression	0.739
	Naive Bayes Classifier	0.727
	Random Forest	0.712
	Network Configuration	0.737
	Deep Learning	0.74

Some commonly used ML models were also fit to the database, using standard specifications. Among those, deep learning [16] using a convolutional neural network (CNN) resulted in the best performance. Specifically, a three-layered fully connected CNN was applied to this dataset and attained an AUROC of 0.74. The XPARS method not only outperforms these models on the ICS data but also has the advantage of being a white box model, in contrast to the typically black box ML models.

Overall, using the ICS dataset, it was shown than the proposed method could predict CVD risk scores relatively accurately using a small chart, hence improving on existing risk assessment methods. The main advantage of this method is that it can provide competitive risk scores even without using a cholesterol measurement. This makes the application of the resulting charts much easier since cholesterol measurement requires a blood test which is expensive, especially in some rural areas.

## Discussion

CVD is the leading cause of death in the world, taking about 17.8 million lives every year [17]. There are general ways to prevent heart disease using statistical or computational techniques. Experiments require advanced equipment that may not be available in remote areas. Therefore, computational methods can be a low-cost and highly accurate substitute for predicting CVD [18]. ML methods are more accurate than conventional statistical methods for predicting the disease [19] since they can account for complex nonlinear relationships between features [20, 21]. However, there is an inherent trade-off between accuracy and interpretability, and the current ML methods applications to CVD risk scores tend to overlook one in favour of the other [22].

ML models can be divided into two categories based on interpretability: white box and black box [23, 24]. While black boxes have high accuracy, they cannot provide a clear explanation of why the prediction was made [25]. On the other hand, white boxes have low accuracy but a clearer interpretation [26, 27]. In many technological and business applications, there is a higher emphasis on prediction accuracy than interpretability, which is generally not the case for medical practice [28]. Physicians, as well as patients, at any stage of counselling and treatment, should be able to trust the model’s interpretability [29], which rules out black box systems [27]. Physicians need to attribute the predicted risk to particular features to address the underlying causes of higher CVD risk. This attribution is possible in white box models such as GA [30, 31]. In this study, GA was developed to build a chart model and improve the results, providing a clear and interpretable method acceptable to the medical community. A multifaceted framework was proposed to consider comprehensibility in modelling. A high-performance, interpretable learning model, was developed. The main goal was to achieve both the advantages of black and white box models to create an interpretive classification with better classification performance compared to a single white box model. Our method could produce simpler predictive charts and provide better estimates of CVD risk in 10 years without the need for clinical or laboratory tests such as high-density lipoprotein (HDL) measurement or blood tests. The improved charts are a population-based study in Iran and could serve as a useful tool in developing future national guidelines for the primary prevention of cardiovascular disease. Health and life expectancy are indicators of the development of societies and countries, so all countries are trying to improve the living standards and health of society.

### Strengths

A novel method was developed to predict CVD risk in an easy-to-use manner accurately. Applying it to the ICS data for the Iranian population, the calibrated XPARS charts could improve existing models based on interpretability or prediction accuracy. XPARS can attain a competitively high prediction accuracy with a small chart, simple to use for both physicians and non-physicians. The main advantage of this model is that it performs very well even in the absence of laboratory access for blood tests, making it easy and cheap to use in many low-income and middle-income countries, even in remote areas. A larger XPARS chart was also calibrated, which could provide more accurate predictions compared to previous models. But as is the case for similarly performing models, the larger XPARS chart requires laboratory access and looking up the score in a more complex chart.

### Limitation

In this study, there were 5,432 participants, of which only 700 people had a positive class label, which leads to an imbalanced data set. Moreover, the population are from a specific area in Iran, making it difficult to generalize the results to other parts of the world. However, the data coverage could not be improved further without a long-term data collection, given this is the most comprehensive dataset for CVDs in the Iranian population. In terms of the methodology, the developed GA method trained one block of the chart at a time. As a result, XPARS with eight risk factors was trained on 160 blocks. Given the granularity of the chart, only 107 blocks out of 160 had at least one observation, and only 20 had more than 100 data points. The model performed well given this data limitation, but more data could have potentially increased model performance by far.

### Future implications

In future research, the characteristics of intervals such as BP can also be considered and coded so that the algorithm itself can find its values. The foundation was laid in the age range, BP and cholesterol of the PARS model and made a model according to which the intervals can be coded, and its calculation can be assigned to the algorithm in such a way that it was possible to weigh a specific range in a chart showed more details, such as heart disease at a younger age, where details are more important; This approach should be considered in the construction of the chart in other populations, and the new charts of the World Health Organization and other countries should be calculated in this way.

## Supporting information

S1 Fig (TIF)

S1 File (DOCX)

## References

[1] Goff DC, Lloyd-Jones DM, Bennett G, Coady S, D’agostino RB, Gibbons R, et al. 2013 ACC/AHA guideline on the assessment of cardiovascular risk: a report of the American College of Cardiology/American Heart Association Task Force on Practice Guidelines. Journal of the American College of Cardiology. 2014;63(25 Part B):2935–59. doi: 10.1161/01.cir.0000437741.48606.98 24239921 PMC4700825

[2] Guo Y, Miao C, Bao M, Xing A, Chen S, Wu Y, et al. Cardiovascular Health Score and the Risk of Cardiovascular Diseases. Plos One. 2015;10(7). doi: 10.1371/journal.pone.0131537 26154254 PMC4495991

[3] Malcolm S, Dorvil M, Zou B, DeGennaro V. Estimating 10-year cardiovascular disease risk in urban and rural populations in Haiti. Clinical Epidemiology and Global Health. 2020;8(4):1134–9. doi: 10.1016/j.cegh.2020.04.004

[4] Bajpai V. The Challenges Confronting Public Hospitals in India, Their Origins, and Possible Solutions. Advances in Public Health. 2014;2014:898502. doi: 10.1155/2014/898502

[5] Lagerweij GR, Moons KGM, de Wit GA, Koffijberg H. Interpretation of CVD risk predictions in clinical practice: Mission impossible? PLoS One. 2019 Jan 9;14(1):e0209314. doi: 10.1371/journal.pone.0209314 ; PMCID: PMC6326414. 30625177 PMC6326414

[6] Mendis S, Lindholm LH, Mancia G, Whitworth J, Alderman M, Lim S, et al. World Health Organization (WHO) and International Society of Hypertension (ISH) risk prediction charts: assessment of cardiovascular risk for prevention and control of cardiovascular disease in low and middle-income countries. J Hypertens. 2007;25(8):1578–82. Epub 2007/07/11. doi: 10.1097/HJH.0b013e3282861fd3 . 17620952

[7] D’Agostino RB, Vasan RS, Pencina MJ, Wolf PA, Cobain M, Massaro JM, et al. General Cardiovascular Risk Profile for Use in Primary Care. Circulation. 2008;117(6):743–53. doi: 10.1161/CIRCULATIONAHA.107.699579 18212285

[8] Assmann G, Cullen P, Schulte H. Simple scoring scheme for calculating the risk of acute coronary events based on the 10-year follow-up of the prospective cardiovascular Munster (PROCAM) study. Circulation. 2002;105(3):310–5. doi: 10.1161/hc0302.102575 11804985

[9] Ridker PM, Buring JE, Rifai N, Cook NR. Development and validation of improved algorithms for the assessment of global cardiovascular risk in women: the Reynolds Risk Score. Jama. 2007;297(6):611–9. Epub 2007/02/15. doi: 10.1001/jama.297.6.611 . 17299196

[10] Sarrafzadegan N, Hassannejad R, Marateb HR, Talaei M, Sadeghi M, et al. PARS risk charts: A 10-year study of risk assessment for cardiovascular diseases in Eastern Mediterranean Region. Plos One. 2017;12(12). doi: 10.1371/journal.pone.0189389 29261727 PMC5736201

[11] Conroy RM, Pyörälä K, Fitzgerald AP, et al. Estimation of ten-year risk of fatal cardiovascular disease in Europe: the SCORE project. Eur Heart J. 2003;24(11):987–1003. doi: 10.1016/s0195-668x(03)00114-3 12788299

[12] Tjoa E. and Guan C., "A Survey on Explainable Artificial Intelligence (XAI): Toward Medical XAI," in IEEE Transactions on Neural Networks and Learning Systems, vol. 32, no. 11, pp. 4793–4813, Nov. 2021, doi: 10.1109/TNNLS.2020.3027314 33079674

[13] Arrieta AB, Díaz-Rodríguez N, Del Ser J, Bennetot A, Tabik S, Barbado A, et al. Explainable Artificial Intelligence (XAI): Concepts, taxonomies, opportunities and challenges toward responsible AI. Information fusion. 2020;58:82–115. doi: 10.1016/j.inffus.2019.12.012

[14] Talaei M, Sarrafzadegan N, Sadeghi M, Oveisgharan S, Marshall T, Thomas GN, et al. Incidence of cardiovascular diseases in an Iranian population: the Isfahan Cohort Study. Arch Iran Med. 2013;16(3):138–44. Epub 2013/02/26. . 23432164

[15] Moshayedi AJ, Gheibollahi M, Liao L. The quadrotor dynamic modeling and study of meta-heuristic algorithms performance on optimization of PID controller index to control angles and tracking the route. IAES International Journal of Robotics and Automation. 2020;9(4):256. doi: 10.11591/ijra.v9i4.pp256-270

[16] Moshayedi AJ, Roy AS, Kolahdooz A, Shuxin Y. Deep Learning Application Pros and Cons Over Algorithm. EAI Endorsed Transactions on AI and Robotics. 2022;1:1–13. /doi.org/10.4108/airo.v1i.19

[17] Wu X, Zhu B, Xu S, Bi Y, Liu Y, Shi J. A cross country comparison for the burden of cardiovascular disease attributable to tobacco exposure in China, Japan, USA and world. BMC Public Health. 2020;20(1):888. doi: 10.1186/s12889-020-09031-7 32513150 PMC7282071

[18] Niederer SA, Lumens J, Trayanova NA. Computational models in cardiology. Nature Reviews Cardiology. 2019;16(2):100–11. doi: 10.1038/s41569-018-0104-y 30361497 PMC6556062

[19] Liu B, Weng SF, Reps J, Kai J, Garibaldi JM, Qureshi N. Can machine-learning improve cardiovascular risk prediction using routine clinical data? Plos One. 2017;12(4). doi: 10.1371/journal.pone.0174944 28376093 PMC5380334

[20] Berglund E, Lytsy P, Westerling R. Adherence to and beliefs in lipid-lowering medical treatments: a structural equation modeling approach including the necessity-concern framework. Patient Education and Counseling. 2013;91(1):105–12. doi: 10.1016/j.pec.2012.11.001 23218590

[21] Dreiseitl S, Ohno-Machado L. Logistic regression and artificial neural network classification models: a methodology review. Journal of biomedical informatics. 2002;35(5–6):352–9. doi: 10.1016/s1532-0464(03)00034-0 12968784

[22] Dimopoulos AC, Nikolaidou M, Caballero FF, Engchuan W, Sanchez-Niubo A, Arndt H, et al. Machine learning methodologies versus cardiovascular risk scores, in predicting disease risk. BMC Medical Research Methodology. 2018;18(1). doi: 10.1186/s12874-018-0644-1 30594138 PMC6311054

[23] Doshi-Velez F, Kim B. Towards A Rigorous Science of Interpretable Machine Learning. arXiv: Machine Learning. 2017. doi: 10.48550/arXiv.1702.08608

[24] Fernandez A, Herrera F, Cordon O, Jose del Jesus M, Marcelloni F. Evolutionary Fuzzy Systems for Explainable Artificial Intelligence: Why, When, What for, and Where to? IEEE Computational Intelligence Magazine. 2019;14(1):69–81. doi: 10.1109/mci.2018.2881645

[25] Rudin C, Radin J. Why Are We Using Black Box Models in AI When We Don’t Need To? A Lesson From An Explainable AI Competition. Harvard Data Science Review. 2019;1(2). doi: 10.1162/99608f92.5a8a3a3d

[26] Carvalho DV, Pereira EM, Cardoso JS. Machine Learning Interpretability: A Survey on Methods and Metrics. Electronics. 2019;8(8). doi: 10.3390/electronics8080832

[27] Lipton ZC. The Mythos of Model Interpretability: In machine learning, the concept of interpretability is both important and slippery. Queue. 2018;16(3):31–57. doi: 10.1145/3236386.3241340

[28] Guidotti R, Monreale A, Ruggieri S, Turini F, Giannotti F, Pedreschi D. A Survey of Methods for Explaining Black Box Models. ACM Computing Surveys. 2019;51(5):1–42. doi: 10.1145/3236009

[29] Lauritsen SM, Kristensen M, Olsen MV, Larsen MS, Lauritsen KM, Jørgensen MJ, et al. Explainable artificial intelligence model to predict acute critical illness from electronic health records. Nature Communications. 2020;11(1). doi: 10.1038/s41467-020-17431-x 32737308 PMC7395744

[30] Mishra DB, Acharya AA, Acharya S. White Box Testing Using Genetic Algorithm—An Extensive Study. A Journey Towards Bio-inspired Techniques in Software Engineering: Springer; 2020. p. 167–87. doi: 10.1007/978-3-030-40928-9_9

[31] N. Al Moubayed and A. Windisch, "Temporal White-Box Testing Using Evolutionary Algorithms," 2009 International Conference on Software Testing, Verification, and Validation Workshops, 2009, pp. 150–151, doi: 10.1109/ICSTW.2009.17

