# Supplementary figures and images for: An evolutionary machine learning algorithm for cardiovascular disease risk prediction
Source: PLoS One. 2022 Jul 28;17(7):e0271723. doi: 10.1371/journal.pone.0271723 (PMC9333440; doi:10.1371/journal.pone.0271723)

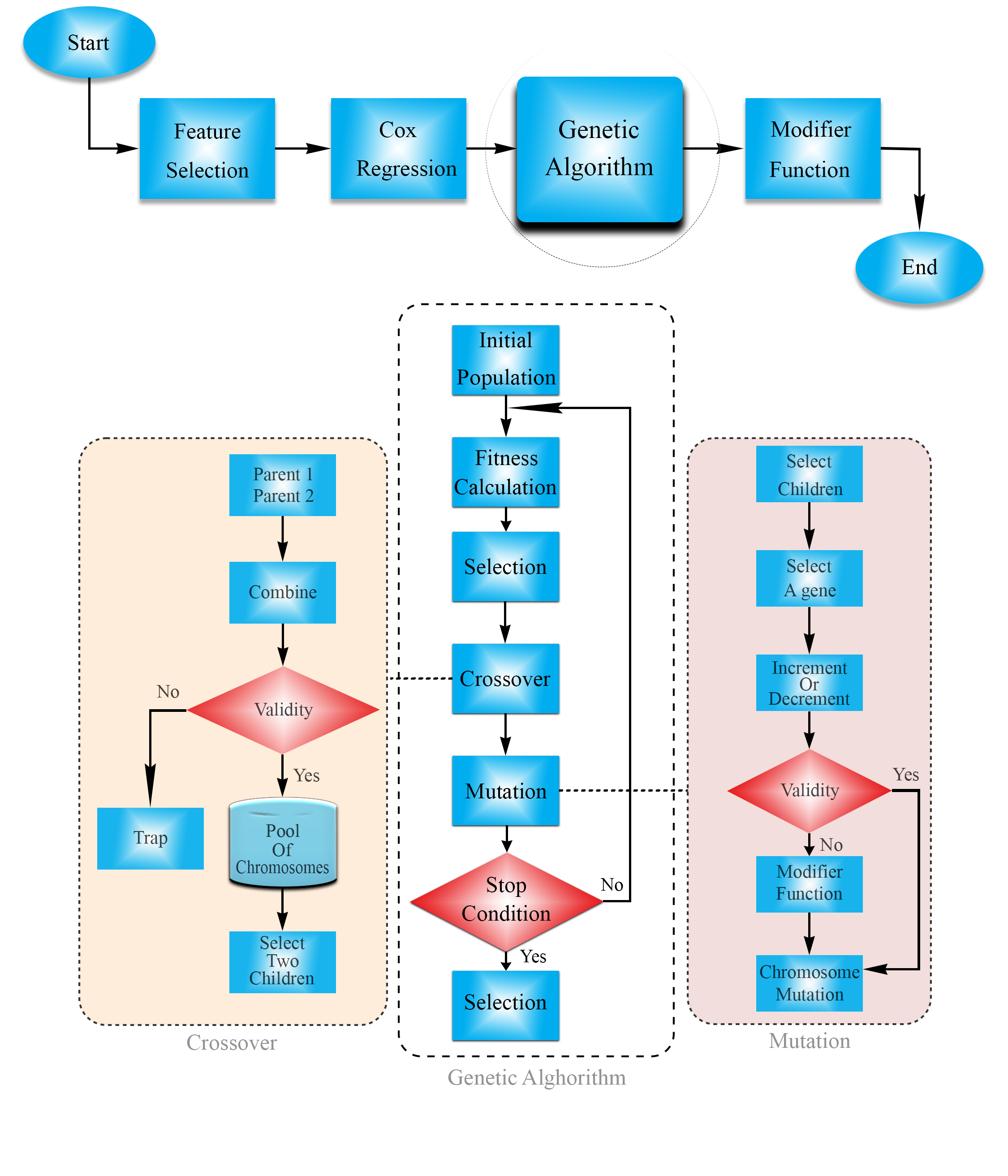

Supplement: S1 Fig — (TIF) [file pone.0271723.s001.tif]
