# Supplementary material for: An evolutionary machine learning algorithm for cardiovascular disease risk prediction
Source: PLoS One. 2022 Jul 28;17(7):e0271723. doi: 10.1371/journal.pone.0271723 (PMC9333440; doi:10.1371/journal.pone.0271723)
Supplement: S1 File — (DOCX) [file pone.0271723.s002.docx]

**An Evolutionary Machine Learning Algorithm for Cardiovascular Disease Risk Score Prediction**

Mohammad Ordikhani^1^, Mohammad Saniee Abadeh^1*^, Christof Prugger^2^, Razieh Hassannejad^3^, Noushin Mohammadifard^4^, Nizal Sarrafzadegan^5, 6^*

^1^ Faculty of Electrical and Computer Engineering, Tarbiat Modares University, Tehran, Iran; ^2^ Institute of Public Health, Charité - Universitätsmedizin Berlin, Berlin, Germany; ^3^ Interventional Cardiology Research Center, Cardiovascular Research Institute, Isfahan University of Medical Sciences, Isfahan, Iran;^4^ Hypertension Research Center, Cardiovascular Research Institute, Isfahan University of Medical Sciences, Isfahan, Iran; ^5^ Isfahan Cardiovascular Research Center, Cardiovascular Research Institute, Isfahan University of Medical Sciences, Isfahan, Iran; ^6^ School of Population and Public Health, Faculty of Medicine, University of British Columbia, Vancouver, British Columbia, Canada.

* Corresponding author: Mohammad Saniee Abadeh. [saniee@modares.ac.ir](mailto:saniee@modares.ac.ir)

*Corresponding author: Nizal Sarrafzadegan. [nsarrafzadegan@gmail.com](mailto:nsarrafzadegan@gmail.com), nizal.sarrafzadegan@ubc.ca


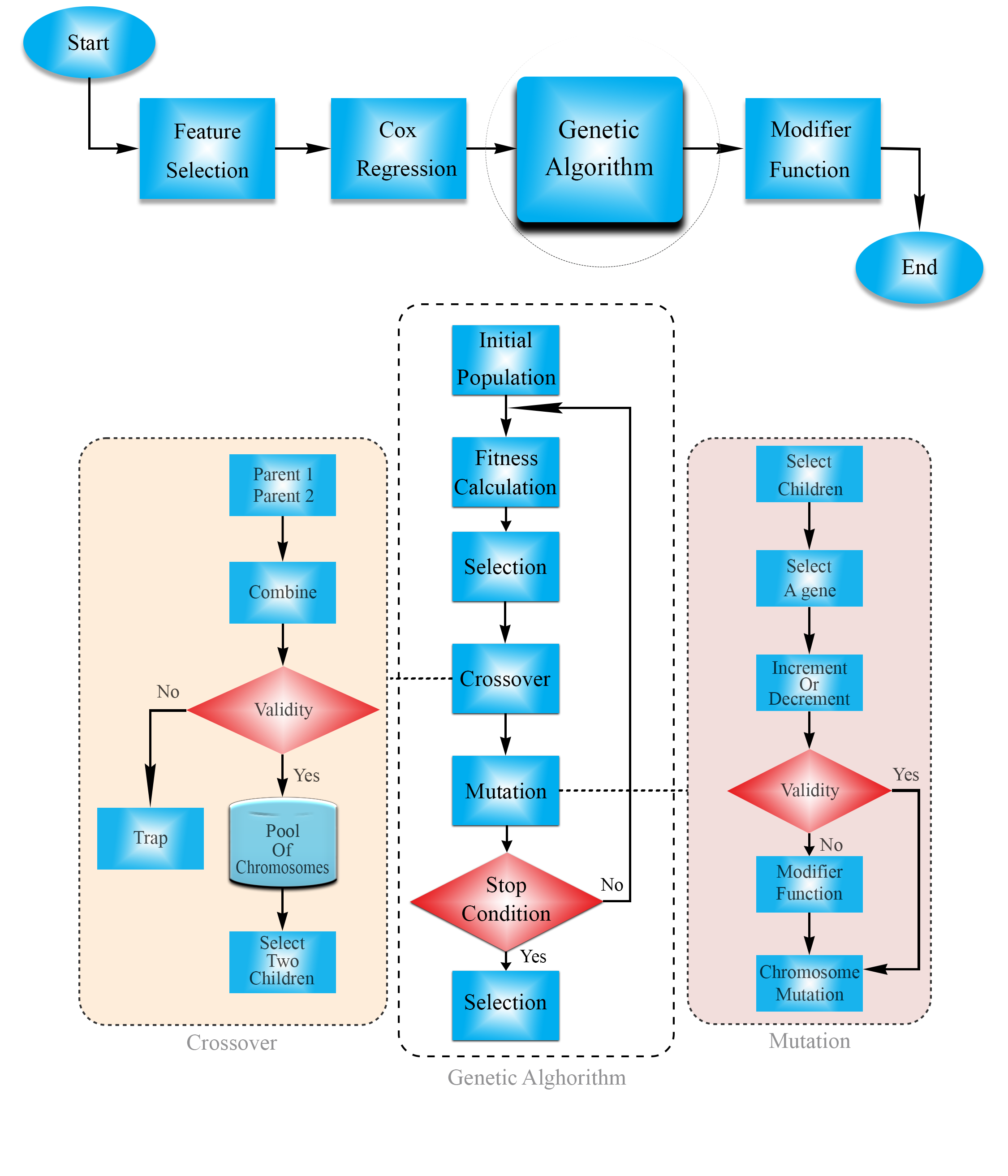


**Fig S1. General details of the algorithm**

**Table S1. CVD risk factors using Cox regression model for the ICS 10-year follow-ups from 2001 to 2011.**

| Mean | Hazard Ratio | Estimate | Risk factors | |
| --- | --- | --- | --- | --- |
| 50.691797 | 1.038 | 0.03759 | Age | |
| 0.487189 | 1.335 | 0.28957 | Male | |
|  |  | - | <=150 | Cholesterol |
| 0.326820 | 1.231 | 0.20759 | 150–200 |  |
| 0.352811 | 1.408 | 0.34201 | 200–250 |  |
| 0.164424 | 1.573 | 0.45316 | 250–300 |  |
| 0.061935 | 1.731 | 0.54847 | >=300 |  |
|  | - | - | <=120 | Blood pressure |
| 0.353548 | 1.578 | 0.45643 | 120–139 |  |
| 0.126820 | 2.09 | 0.73697 | 140–159 |  |
| 0.072995 | 2.848 | 1.0467 | >=160 |  |
| 0.110046 | 1.878 | 0.63041 | Diabetes | |
| 0.675945 | 1.31 | 0.26989 | High waist to hip ratio | |
| 0.053272 | 1.495 | 0.40182 | Family history of CVD | |
| 0.219724 | 1.336 | 0.28974 | Smoking | |
